# Directed Gaze Trajectories for Biometric Presentation Attack Detection

**DOI:** 10.3390/s21041394

**Published:** 2021-02-17

**Authors:** Asad Ali, Sanaul Hoque, Farzin Deravi

**Affiliations:** School of Engineering and Digital Arts, University of Kent, Canterbury CT2 7NT, UK; asad_a5@yahoo.com (A.A.); F.Deravi@kent.ac.uk (F.D.)

**Keywords:** biometrics, face recognition, presentation attack detection, sensor-level spoofing, gaze tracking

## Abstract

Presentation attack artefacts can be used to subvert the operation of biometric systems by being presented to the sensors of such systems. In this work, we propose the use of visual stimuli with randomised trajectories to stimulate eye movements for the detection of such spoofing attacks. The presentation of a moving visual challenge is used to ensure that some pupillary motion is stimulated and then captured with a camera. Various types of challenge trajectories are explored on different planar geometries representing prospective devices where the challenge could be presented to users. To evaluate the system, photo, 2D mask and 3D mask attack artefacts were used and pupillary movement data were captured from 80 volunteers performing genuine and spoofing attempts. The results support the potential of the proposed features for the detection of biometric presentation attacks.

## 1. Introduction

Despite their widespread use and acceptance in an ever-increasing range of applications, biometric person recognition systems remain vulnerable to sophisticated spoofing attacks that can undermine the trust in them [1]. This type of spoofing is a direct attack on the sensor and is also known as presentation attack. Such presentation attacks use artefacts such as photos or masks that may be created from the previously captured data of genuine users and then presented at the system sensor(s). In this way, without any or much prior knowledge about the internal operation of the biometric system, a fake biometric sample of a genuine user can be presented by an impostor to gain unauthorised access. To detect such sensor-level attacks, it is necessary to automatically recognise if any artefacts are being used and establish that a genuine user is present at the sensor providing the “live” sample.

This paper presents novel features, based on stimulated pupillary movements, for presentation attack detection (PAD), extending our work originally reported in [2]. The users’ gaze is directed along a random path presented via a display device. The accuracy with which this path is followed by the users’ gaze is then used as a means of detecting presentation attacks; the underlying assumption being that the use of an attack artefact such as a photo or mask by an imposter makes it more difficult to follow the path of the stimulus accurately. Different stimulus trajectories, durations, planar geometries and attack artefacts are evaluated through data captured from 80 volunteers. The results are discussed and compared with other approaches for presentation attack detection. While our previous work [3] was able to deal with video attacks, the scope of this work is limited to photo and mask attack detections only. The proposed approach does not protect against video replay attack. The focus of this work is the reduction in the user interaction time required for the mask and photo attack instruments while considering a more restrictive device geometry to simulate a mobile device application.

The paper is organised as follows. Section 2 provides an overview of the state-of-the-art related to presentation attack detection. Section 3 describes the proposed techniques including two different types of challenge trajectories, and the feature extraction technique. Section 4 presents the evaluation protocol and the experimental results and finally Section 5 provides conclusions and suggestions for further work.

## 2. State of the Art

A wide range of different approaches have been reported by researchers for biometric presentation attack, or alternatively “liveness” detection, which may be categorised as “passive” and “active”. Passive approaches are characterised by not requiring the cooperation of users or even their conscious participation in the detection process. They may exploit involuntary physical movements, such as spontaneous eye movements, or static features that may indicate the presence and use of an artifact. By contrast, active approaches require the engagement of the user with the detection process.

### 2.1. Passive Techniques

Eye blinks have been used as a means of human–computer interaction [4,5]. Being a natural bodily function, the opening and closing of the eyelid has also been used for the biometric presentation attack detection. Lin Sun et al. [6,7] used conditional random fields (CRFs) for eye blink detection as a means of establishing face liveness. Temporal information from the stages of the eye-blink process were extracted and was used to determine liveness.

In photo attacks based on printing a photograph of a genuine user on paper as artefact, some texture attributes may be introduced which are not present in the images when captured directly from the genuine users’ faces. Schwartz et al. [4] used such a passive approach for photo attack detection based on a combination of texture, colour and shape information. Feature vectors thus formed combined the low-level feature descriptors comprising spatial and temporal information and used partial least squares regression to differentiate between the genuine and spoof images or videos. Experiments were carried out to validate their method with datasets of still images and videos. They used the NUAA dataset and the experiments showed that the methods were effective for photo attack detection.

Similarly, in video replay attacks it has been reported that distinctive features commonly appear in the information acquired by the biometric sensor which can be used for attack detection. Pinto et al. [5] analysed the noise characteristics which are generated during such attacks. The Fourier transform was used to probe the captured videos to extract such noise properties for presentation attack detection.

Nguyen et al. [8] also introduced a parametric approach for face PAD using a statistical model of image noise for the skin regions of the face. Images from the photo attack artefacts (either printed or projected images) reveal specific textural information caused by the presentation process which make these different from images captured from genuine presentations. Noise model parameters were derived only from genuine presentations. Their proposed system outperformed the selected benchmarking reference systems when the attack types were unknown.

Using LBP-based micro texture analysis, Maatta et al. [9] recommended an approach centred on reflective characteristics of different objects. The differences between genuine and printed photos reveal that genuine and printed photos reflect light differently. Face prints may consist of jitter and banding which can be detected with texture and local feature analysis. In their work, facial images were divided into several local regions and three descriptors were extracted from each block. The LBP operator was applied on the normalised face image.

The three-dimensional nature of genuine facial presentations at the biometric sensors is another source of information for detecting photo and video attacks. The 3D facial structure was used by Lagorio et al. [10] for presentation attack. Their proposed approach could be deployed in either 2D or 3D face recognition systems to detect spoofing attacks.

### 2.2. Active Techniques

The category of active techniques for the presentation attack detection includes systems based on a challenge–response interaction with the user. In such systems, the user is asked to perform specific tasks to establish their presence and intentional engagement with the biometric process. Examples of such tasks include speaking specific words or phrases or moving parts of the body.

Mouth movements along with eye movements were used by Singh et al. [11] for presentation attack detection. The random generation of challenge sequences was used to prevent the success of sophisticated presentation attacks.

The use of controlled illumination can also help in detecting presentation attacks. Smith et al. [12] proposed an approach to counter replay attacks on smart devices using different screen colours to illuminate the user’s face. The corresponding reflections from the face due to these random colours were then analysed to determine if an attack artefact was present.

Frischholz et al. [13] explored a challenge–response approach where the users were given instructions to randomly look in certain directions. The system estimated the head pose of the user and compared it to the instructions given by the system. These estimations were used to classify the genuine and presentation attacks.

The direction of gaze can be a rich source of information for presentation attack detection. Ali et al. [14] was the first to explore novel gaze-based approaches to detect presentation attacks. Ali et al. [2,14,15,16,17,18,19] have subsequently presented a number of novel gaze-based approaches to presentation attack detection. A visual target with randomly assigned trajectories were shown on the display screen for the user to follow with eye (and head) movements. Extracted features from the captured images mainly evaluated the consistency of the eye movements to estimate the liveness of the source. They also explore the impact of tinted glasses on such gaze-based spoofing detection.

Cai et al. [20] also reported a gaze-based system where the user is required to look at points placed on a display screen. For each user a gaze estimation model was trained. The difference between the predicted and the measured points provided the information needed for detecting presentation attacks.

There have also been several survey papers [21,22,23] published recently which can provide a more comprehensive coverage of the state of the art of presentation attack detection research for face biometric systems.

Several datasets [24,25,26,27,28,29,30,31,32,33] have been used to test, evaluate, and compare face–PAD methods. However, the scenarios addressed in the available public databases are mostly photo and video attacks. With the development of 3D printing technologies, 3D masks are becoming a more effective way for presentation attacks. Most 3D PAD datasets are based on wearable hard-resin or silicone masks. Wax figure face databases were reported in [34,35] for super-realistic 3D presentation attack research. For the work presented in this paper, a database has been locally collected as none of the public databases include the type of challenge–response, artefacts and device formats, which are investigated in this paper.

This paper extends the work in [2] by exploring different types of randomised challenge trajectories. It investigates the impact of challenge type and duration on the accuracy of attack detection for different device form factors. This particular feature was based on the relative movement of the pupil within the eye socket. Details of the implementation are described in the following Section.

## 3. Proposed Technique

**Figure 1:**
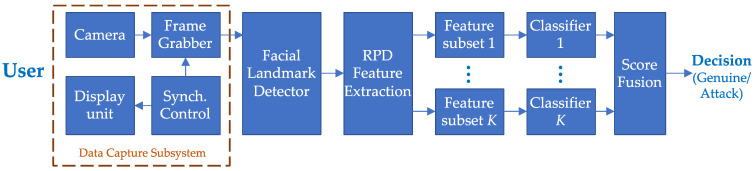
The block diagram of the proposed presentation attack detection (PAD) system. [RPD = Relative Pupillary Displacement].

Figure 1 shows a block diagram describing the main components of the proposed system. A graphical stimulus on a visual display explicitly directs the eye movements of the user and the camera (sensor) which captures a series of facial images during this process. For the experiments reported in the following section, the visual stimulus was a cross shape moving along a set of randomly generated paths at uniform speed. The system extracts facial landmarks from the captured frames. These landmarks, such as eye corners, are then used to compute various features, which are then used to detect whether a presentation attack, using an attack artefact, has been attempted.

### 3.1. Challenge Trajectories

**Figure 2:**
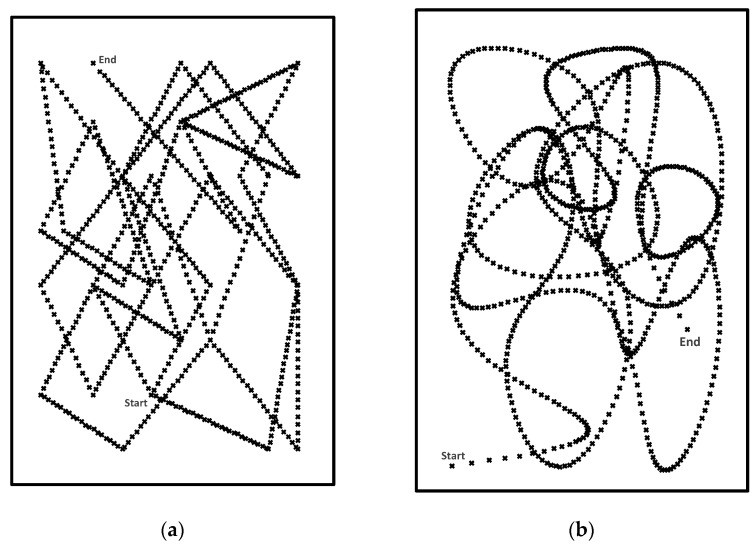
Samples of the random trajectory used as a challenge during the data collection: (**a**) *Lines*; and (**b**) *Curves* (the labels’ “start” and “end” are added for the clarity of illustration only).

In this work, two different challenge trajectories were used to direct the eye movements of the users: *Lines* and *Curves* as illustrated in Figure 2. The start and end points are randomly chosen for each presentation. The representation in Figure 2 illustrates a trace of the stimulus at the end of the presentation. During the presentation, only the stimulus as a small moving shape (‘x’) was visible to the user to elicit their natural head–eye movements.

The *Lines* challenge trajectory is composed of a set of connected straight lines along which the graphical stimulus moves at a constant speed. Each attack detection session may contain a number of line segments with an average of 1 s per line.

The *Curves* challenge has a very similar design to the *Lines* challenge, except that the stimulus moves along randomised curved paths. The curved paths are generated by fitting a spline function on a number of randomly generated control points. The *Curves* challenge thus provides an alternative stimulus to encourage the smooth pursuit of gaze.

In a practical application, it is anticipated that only a short presentation of the stimulus (of the order of a few seconds) may be sufficient to detect presentation attacks. However, the data collected from volunteers were of much longer duration for the purpose of evaluations. These data were then partitioned into shorter segments and used in experiments to establish the trade-off between the duration and attack detection accuracy. This longer data capture duration may also provide useful data for assessing the impact of fatigue on the effectiveness of the system as well as any learning effects by attackers that may improve their threat potential.

### 3.2. Facial Landmark Detection, Feature Extraction and Classification

The Chehra Version 3.0 [36] software was used to process the facial images in order to extract up to 59 facial landmarks. Features were extracted in the proposed scheme using the coordinates of the landmarks around the eye regions as described below.

**Figure 3:**
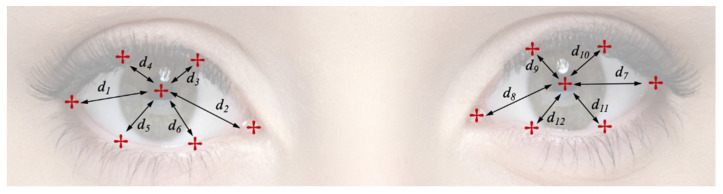
Detected Landmarks and corresponding distances from the pupil centres used for feature extraction. Features from the two eyes were treated independently for attack detection in this implementation.

Figure 3 shows the periocular landmarks extracted from each eye. The distances of these points from the corresponding pupil centres form the basis of the proposed feature. The key motivations behind such features were that they capture eye movements which are less impacted by head movement/pose and when normalised, are not affected by scale, tilt, etc.

Let R be a set of these landmarks from a series of ‘N’ facial images acquired during a user interaction:

(1) $$R=\left\{r_{1},r_{2},\cdots ,r_{n},\cdots ,r_{N}\right\},\;r_{n}=\left\{\left(u_{n_{i}},v_{n_{i}}\right)\right\},$$

where $\left(u_{n_{i}},v_{n_{i}}\right)$ is the *i*th landmark location of the *n*th image frame, and ‘*m*’ periocular landmarks along with the pupil centres were extracted here (*i* = [1,*m*]).

For each image frame, a set of ‘*m*’ normalised Euclidean distances were calculated between the centre of the pupil and the landmarks on the corresponding eye socket as:

(2) $$D_{n}=\left\{d_{n_{1}},d_{n_{2}},d_{n_{3}},d_{n_{4}},\ldots ,d_{n_{m}}\right\}.$$

All $D_{n}$ values were normalised by $D_{n_{nor}}$ where $D_{n_{nor}}\;$is the Euclidean distance between the left and right corner of the relevant eye sockets in the image.

(3) $$D=\left[\begin{array}{c} D_{1}\\ D_{2}\\ \vdots \\ D_{n}\\ \vdots \\ D_{N} \end{array}\right]=[\begin{array}{ccccc} d_{1_{1}} & d_{1_{2}} & d_{1_{3}} & & d_{1_{m}}\\ d_{2_{1}} & d_{2_{2}} & d_{2_{3}} & & d_{2_{m}}\\ \vdots & \vdots & \vdots & \cdots & \vdots \\ d_{n_{1}} & d_{n_{2}} & d_{n_{3}} & & d_{n_{m}}\\ \vdots & \vdots & \vdots & & \vdots \\ d_{N_{1}} & d_{N_{2}} & d_{N_{3}} & & d_{N_{m}} \end{array}]$$

The nature of the pupillary motion during the challenge–response exercise can now be modelled from the distribution of these normalised distance values and in this particular study, were represented by the standard statistical attributes such as

(4) $$\begin{array}{c} D_{var}=var\left(D\right)\\ D_{max}=max\left(D\right)\\ D_{min}=min\left(D\right) \end{array}$$

A collection of these then forms the proposed feature vector for presentation attack detection:

(5) $$F=\left[D_{var},\;D_{max},D_{min}\right].$$

In this implementation, separate feature vectors are extracted from each eye and used for attack detection independently. These corresponding outcomes are then combined using the *product* fusion rule for the final decision.

Let there be *C* classifiers each independently processing the different features extracted from different facial landmarks. Each classifier generates class-wise scores (e.g., *a posteriori* probabilities for each of the Ω classes) whether the attempt is genuine or one of the spoof attacks:

(6) $$score_{i}\left(\omega \right)=classifier_{i}\left(F\right),\;1\leq i\leq C,\;1\leq \omega \leq \Omega$$

In this implementation, we had two classifiers (*C* = 2) analysing the feature vectors from each eye. The number of classes is also two (‘genuine’ or ‘attack’ presentation).

The ‘product rule’ fusion [37] combines these individuals scores (assuming mutual independence) and the decision is assigned to the class giving the highest score as shown below:

(7) $$Overall\_score\left(\omega \right)=\underset{i}{\prod }score_{i}\left(\omega \right),\;1\leq i\leq C$$

(8) $$Decision={\arg\;\max}_{\omega }\;\left(Overall\_score\right),\;1\leq \omega \leq \Omega$$

## 4. Experimental Evaluation

Three types of attack artefacts were used here in order to evaluate the proposed techniques. The attack scenarios assume an impostor attempting to subvert the biometric system by displaying a high-resolution image of a genuine user on a tablet screen (photo attack), or a high-quality printed colour photo with holes in place of the pupils held in front of the impostor’s face as a mask (2D mask attack) or presenting a three-dimensional mask constructed using the genuine user’s data (3D mask attack) [17].

**Figure 4:**
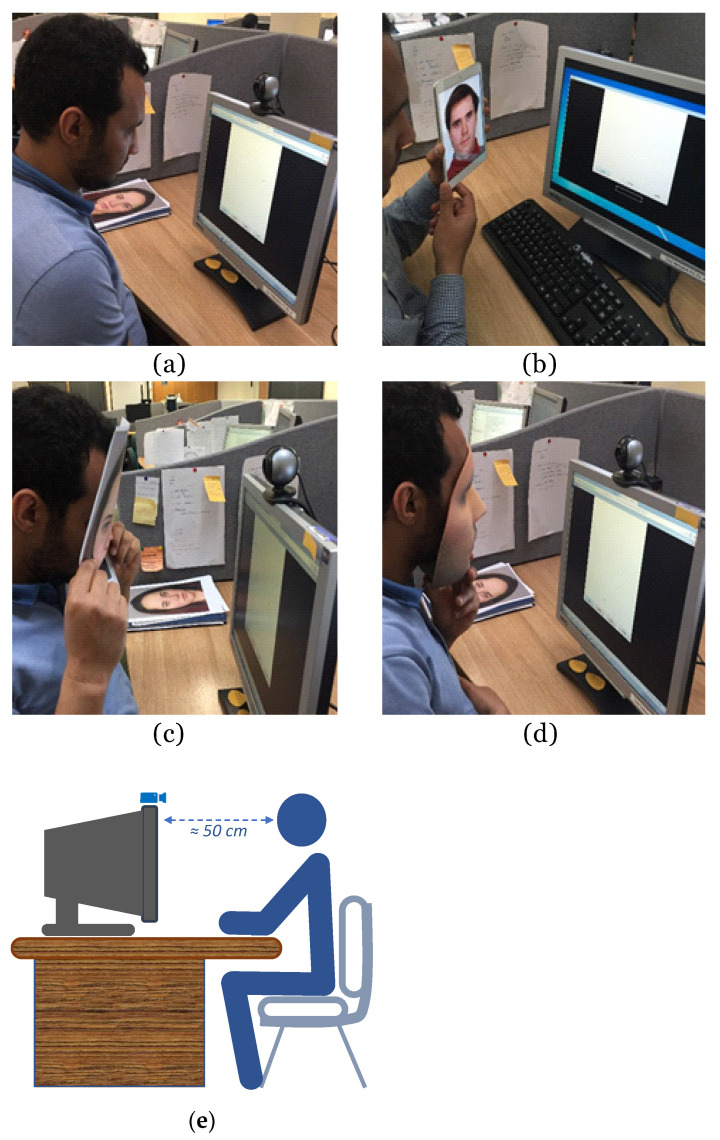
Data collection process: (**a**) genuine attempt; (**b**) photo attack; (**c**) 2D mask attack; (**d**) 3D mask attack; and (**e**) the setup used for data acquisition.

Eighty adult male and female participants from a range of ethnic backgrounds were recruited to evaluate the proposed system while acting as both genuine users and impostors. The number of participants was similar to that used in other published work in the presentation attack detection and should be sufficient to illustrate the potential of the proposed approach. Figure 4 illustrates the hardware setup for data acquisition as well as snapshots of user attempts (both genuine and impostor attacks). Figure 4a is an example of a genuine attempt, Figure 4b shows projected photo attack, and Figure 4c,d show 2D mask and 3D mask attacks, respectively.

Two different device geometries were simulated on a desktop display for system evaluation. Active screen areas of dimensions 6.45 × 11.30 cm^2^ and 15.87 × 21.18 cm^2^ were used, which corresponds to typical handheld mobile phone and tablet devices, respectively. These formats were envisaged the most likely ones that may be used while accessing services through mobile devices. Acquired data were partitioned at the 60:40 ratio for training and test purposes while the *k*-NN schemes were used for PAD classification.

**Figure 5:**
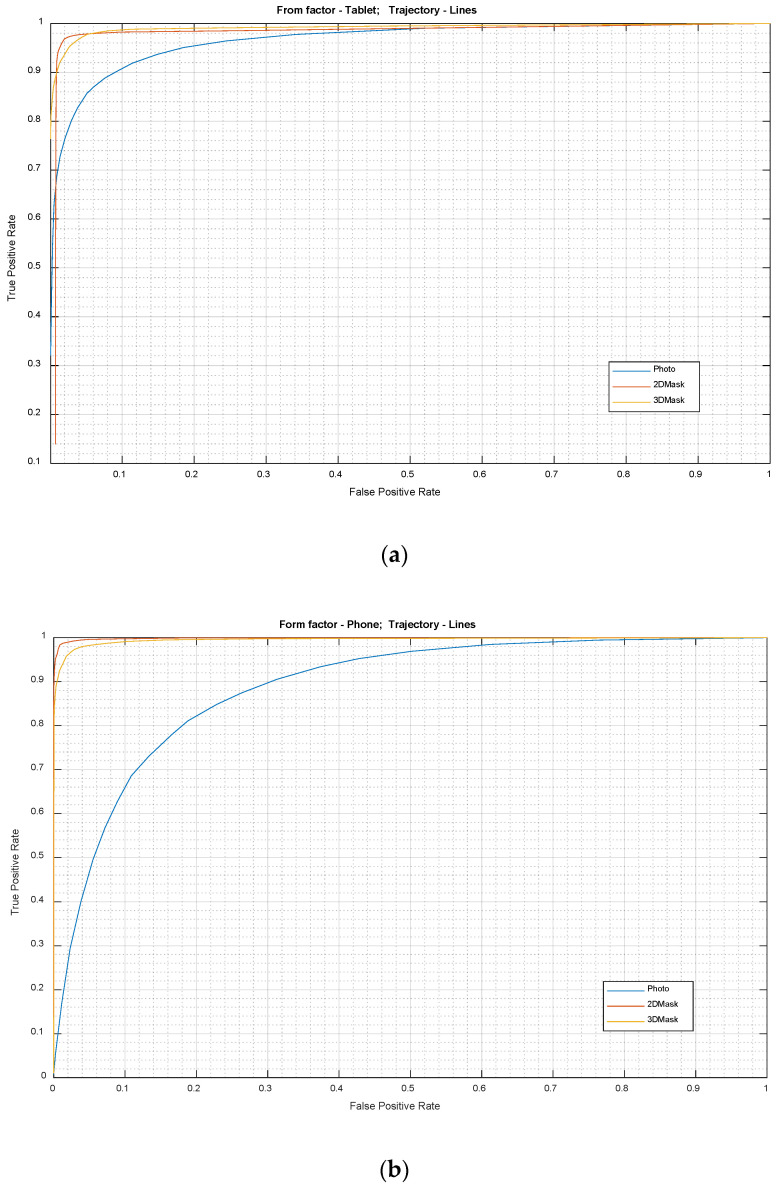
ROC curves for the photo, 2D mask and 3D mask. Stimulus trajectory: *Lines*. Form factors: (**a**) tablet format; and (**b**) phone format.

The Receiver Operating Characteristic (ROC) curves for photo, 2D mask and 3D mask attacks for the tablet format with the *Lines* challenge for attempt durations of 5 s are given in Figure 5a. Here, *True Positive Rate (TPR)* relates to decisions where genuine user attempts are correctly identified whereas *False Positive Rates (FPR)* are presentation attacks not detected by the system. While the 2D and 3D mask attacks are relatively easy to detect using the *Lines* challenge trajectory type, photo attack detections were significantly more difficult. This can be due to the fact that images captured from the photo attacks are smaller in size and of lower quality, making gaze feature extraction more susceptible to noise.

The ROC curves for the three attack scenarios in the phone format are given in Figure 5b. The photo attack again appears to be relatively harder to detect compared to the 2D and 3D mask attacks which are easy to detect. 3D attacks are attacks which are slightly harder to detect compared to 2D mask attacks.

Table 1 provides a summary of the performance figures for the photo, 2D mask and 3D mask attacks both in Tablet and Phone formats at various FPR settings. The system performance values are reasonably high at 10% FPR. However, as the FPR values are lowered, the performance, especially of the photo attack detection, drop significantly at lower FPRs. Especially for the phone format, at 1% FPR, the TPR values dropped to as low as 16%. Nevertheless, in cases of 2D and 3D mask attacks, the reduction in system performance was much smaller compared to the photo in both tablet and mobile format.

Table 2 provides a comparison of the performance of the three PAD cases in both Tablet and Phone formats for various challenge time durations. In most cases, the performance remains almost unchanged for 3, 5 and 10 s challenge durations. This suggests that short duration challenges may be acceptable, thus enhancing the usability of the proposed approach. Performance in photo attack detection (esp. for the phone format) has increased with longer durations, indicating that for some difficult cases, relatively longer challenges can improve the robustness of the system.

The next set of results explore the impact of a challenge scenario comprising smooth pursuits only and report on a set of experiments with data which were captured while using the *Curves* challenge. The purpose of these experiments was to check the effect of the challenge trajectory design on the performance of the system. It is envisaged that the abrupt directional changes present in the *Lines* stimuli may have had detrimental effect (such as trigger large head movements) and the new *Curves* challenge will inspire a smooth pursuit of gaze. Once again, all three attack artefact types, photo, 2D and 3D masks were used and the tablet and phone challenge geometries were investigated.

**Figure 6:**
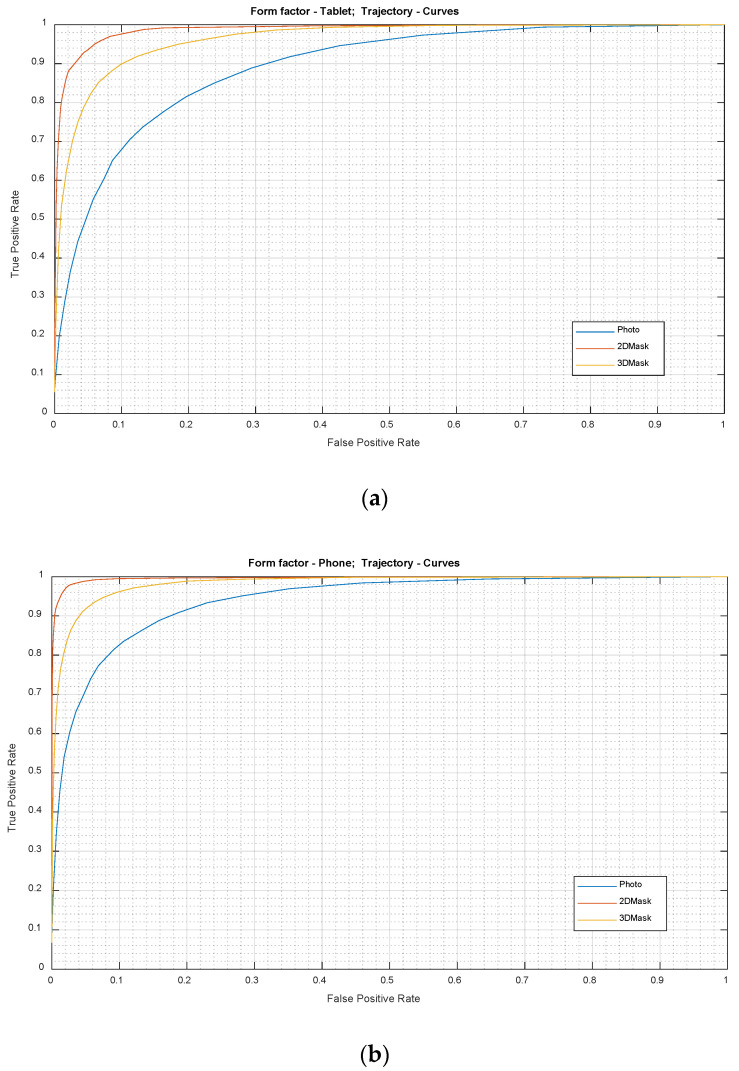
ROC curves for the photo, 2D mask and 3D mask. Stimulus trajectory: *Curves*. Form factors: (**a**) tablet format; and (**b**) phone format.

Figure 6 presents the ROC plots for the *Curves* challenge trajectory for the three attack artefact types. The proposed method again performed very effectively in distinguishing 2D mask attacks from genuine presentation. The performance for the 3D mask attacks, while not as good as that for 2D mask detection, was reasonably close. However, significantly low performance was observed for the photo attack detection for both of the form factors. Table 3 provides a summary of the results for various FPR settings with the *Curves* challenge trajectory. When compared with the *Lines* challenge figures in Table 2, the TPR values for the *Curves* stimulus were relatively lower for most of the attack scenarios except that the photo attack detection rate for the phone format improved. This indicates that there exists some complementarity between these two challenge trajectories and a hybrid one may prove optimal.

The performance of the proposed PAD system using the *Curves* challenge trajectory at 10% FPR is summarised in Table 4 for various challenge durations. Unlike the *Lines* challenge, TPR values improved with the increased challenge duration. In particular, the performance for photo attacks, while lower than that for the other attack types, did noticeably improve with increased challenge duration. The relatively low performance for Photo attacks may be due to the relatively small size of the photos used in the simulated attack, making feature extraction less precise. Even for a 5 s challenge duration, the RPD-based system was able to achieve a TPR accuracy of 90% or more for the two types of mask attack.

**Figure 7:**
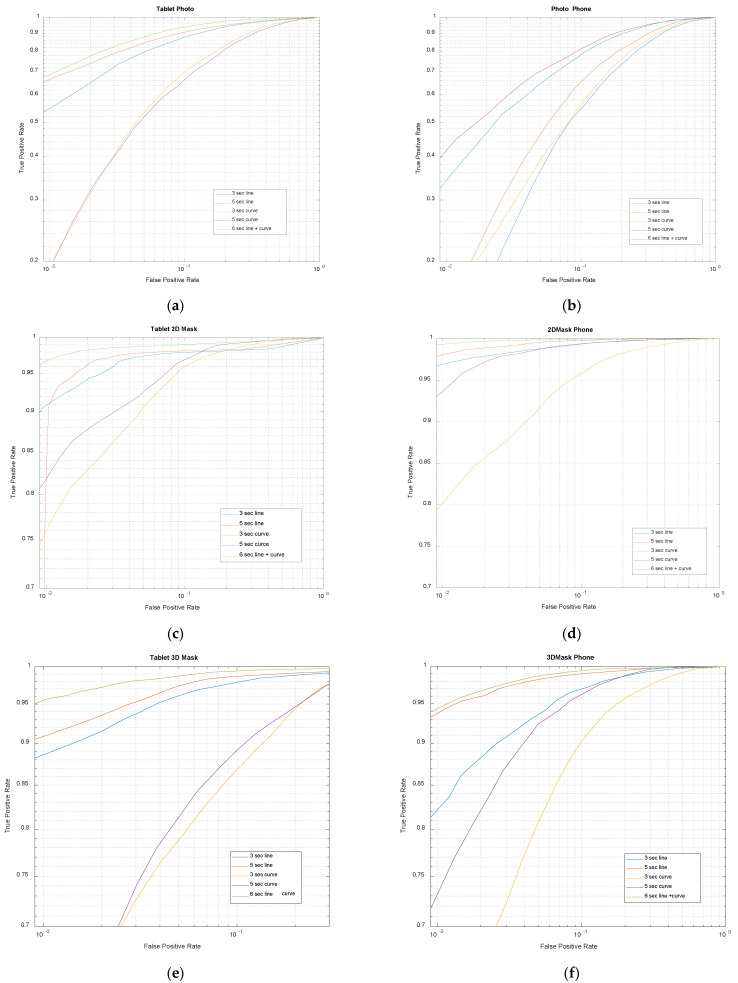
ROC curves for the photo, 2D mask and 3D mask. Stimulus trajectory: composite. Form factors: tablet format (**left**) and phone format (**right**). [The plot titles indicate the device format as well as the attack artefact.]

In the following set of experiments, we explored the feasibility of a composite scheme simulating a hybrid scenario where the user was presented with both visual challenges in succession and the final decision was based on the fusion of the outcome of the two components. Figure 7 shows the results for this composite challenge for the two device formats and three attack types. Each of the challenges were presented to the user for 3 s and were analysed independently before being fused using the product rule. The ROC curves in Figure 7 also include the 3 s- and 5 s-long pure *Lines* and pure *Curves* challenge outcomes for comparison. Logarithmic axes were used to highlight the differences at low FPR settings. It is very obvious that the composite scheme clearly outperformed the individual challenge types by a significant margin, especially for the 2D mask and 3D mask attack types. The response to the photo-attacks was somewhat mixed. For the tablet format, the detection rates were clearly higher than those for the pure *Lines* and pure *Curves* challenges. However, for the phone format, the detection rates were similar to that for the *Curves* challenge albeit a little lower.

Table 5 summarises these performance figures for the photo, 2D mask and 3D mask attacks both in the tablet and phone formats at various FPR settings. It is evident that the TPR values from the composite challenge scenario are noticeably higher than those from the *Lines* or *Curves* challenges only, except for the photo attack detections in the phone format. When compared with the best TPR values obtained (either from the *Lines* or *Curves*), 3–5% improvements can be achieved for the Tablets and 0.5–1.3% for the phone devices at low FPR settings (≤0.03). Even when compared with 10 s pure challenges, in most cases, the 6 s composite challenge performed better.

Only in the photo attack cases on the phone devices, degraded performances (by 5–6%) were noticed. This is most likely due to the low photo attack detection success of the *Lines* challenge in the phone format. Perhaps a careful adjustment in the contribution of *Line* and *Curve* elements in the composite scenario will be able to overcome this anomaly; however, this optimisation has not been explored in this study.

**Figure 8:**
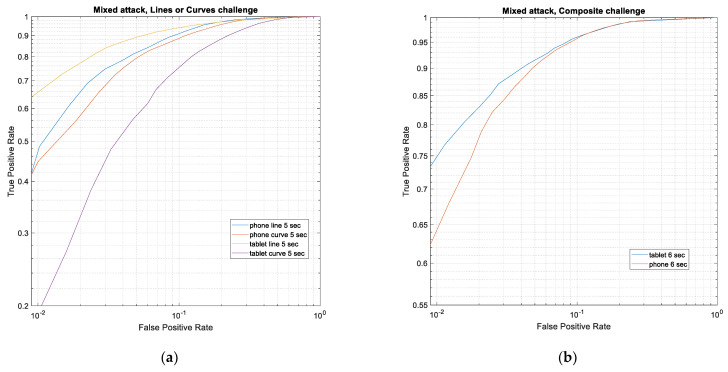
ROC curves for mixed attack types for both device formats. Stimulus trajectory: (**a**) *Lines* or *Curves* challenge for 5 s; (**b**) composite challenge for 6 s.

In a real-life scenario, an impostor may use any of the face artefacts (photo or 2D/3D masks) in their presentation attack. To simulate this, in the following experiment, we combined all the three types of attacks under a single category and assessed the detection success of the proposed system. Figure 8 shows ROC plots for the three challenge scenarios (*Lines*, *Curves*, or composite). The aim here was to detect whether any of the attack artefacts were used or not and no attempt was made to determine the type of artefact. Due to the increased diversity of attack, the TPR values (see Table 6) are lower than those values when the specific attack type was known. However, especially for the Composite challenge type, more than 80% of the attacks were detected at FPR settings of ≥0.02.

Table 7 presents a comparison of performances of PAD techniques reported in the literature with the proposed technique. For this comparison, the results for the proposed system for all three attack types were combined to obtain an overall estimate of the False Negative Rate (FNR) at different FPR settings. As different databases and evaluation protocols were used in the evaluations reported in the literature, it is difficult to make a direct comparison between these results. However, as a general indication of the potential of the proposed eye-movement features for presentation attack detection, the comparison was very promising.

## 5. Conclusions

The work presented in this paper uses eye/pupil movements stimulated by predetermined randomised challenge trajectories to detect biometric presentation attacks. The experimental results support the effectiveness of the proposed features, even when the challenge is presented to users using limited geometries of mobile devices in detecting 2D and 3D mask attacks. The relatively low accuracy in detecting photo attacks may be due to the errors in landmark detection accuracy for low-quality images captured from the photo projection devices used in the experiments. If so, this may be improved by using superior cameras along with robust landmark detection algorithms appropriate for low-resolution images.

The challenge presentation sessions were approximately of 1 min duration but only short segments of up to 10 s were used for feature extraction and system evaluations. The additional data may provide useful information regarding the impact of habituation and fatigue on the effectiveness of the system as well as any learning opportunities for attackers that may improve their threat potential. These aspects would be subject of future studies.

## Figures and Tables

**Table 1:** TPR at various FPR settings (trajectory: *Lines*; duration: 5 s).

Format	Attack Type	TPR (in %)				
		@FPR = 0.01	@FPR = 0.02	@FPR = 0.03	@FPR = 0.05	@FPR = 0.10
Tablet	Photo	69.5	76.4	80.2	85.6	91.0
	2D Mask	93.2	96.8	97.4	97.8	98.2
	3D Mask	90.4	93.6	95.6	97.6	98.7
Phone	Photo	16.1	27.0	35.0	47.0	66.0
	2D Mask	98.5	99.0	99.3	99.6	99.7
	3D Mask	93.2	96.2	97.4	98.2	99.1

**Table 2:** TPR at FPR = 0.10 for various challenge durations (in sec.) (trajectory: *Lines*).

Format	Attack Type	TPR (in %)		
		3	5	10
Tablet	Photo	88.3	91.0	88.4
	2D Mask	98.4	98.2	98.4
	3D Mask	97.9	98.7	97.9
Phone	Photo	58.0	66.0	76.0
	2D Mask	99.3	99.7	99.8
	3D Mask	97.1	99.1	99.1

**Table 3:** TPR at various FPR settings (trajectory: *Curves*, duration: 5 s).

Format	Attack Type	TPR (in %)				
		@FPR = 0.01	@FPR = 0.02	@FPR = 0.03	@FPR = 0.05	@FPR = 0.10
Tablet	Photo	24.0	33.9	41.5	51.5	68.0
	2D Mask	80.0	87.8	90.5	93.6	97.6
	3D Mask	53.0	64.8	72.1	81.0	90.0
Phone	Photo	42.1	55.8	62.5	71.0	83.0
	2D Mask	94.0	97.0	98.0	98.9	99.5
	3D Mask	72.5	82.0	87.0	91.9	96.2

**Table 4:** TPR at FPR = 0.10 for various challenge duration (in sec.) (trajectory: *Curve*).

Format	Attack Type	TPR (in %)		
		3	5	10
Tablet	Photo	69.5	68.0	74.4
	2D Mask	96.8	97.6	99.6
	3D Mask	87.2	90.0	95.2
Phone	Photo	59.4	83.0	86.0
	2D Mask	96.2	99.5	99.4
	3D Mask	91.3	96.2	98.9

**Table 5:** TPR at various FPR settings (trajectory: composite, duration: 3 + 3 s).

Format	Attack Type	TPR (in %)				
		@FPR = 0.01	@FPR = 0.02	@FPR = 0.03	@FPR = 0.05	@FPR = 0.10
Tablet	Photo	68.2	77.1	82.1	88.0	94.0
	2D Mask	96.7	98.2	98.5	98.8	99.0
	3D Mask	95.5	97.2	98.1	98.7	99.4
Phone	Photo	34.0	47.0	55.0	64.8	78.0
	2D Mask	99.3	99.6	99.7	99.8	99.9
	3D Mask	94.4	96.7	97.7	98.7	99.4

**Table 6:** TPR at various FPR settings for mixed attack (trajectory: composite, duration: 6 s).

Format	TPR (in %)				
	@FPR = 0.01	@FPR = 0.02	@FPR = 0.03	@FPR = 0.05	@FPR = 0.10
Tablet	73.5	83.0	87.8	91.5	96.0
Phone	64.1	78.0	84.0	90.5	95.7

**Table 7:** FPR and FNR for various methods.

Method	FPR	FNR
Kollreider et al. [38]	1.5%	19.0%
Tan et al. cf. [39]	9.3%	17.6%
Peixoto et al. [39]	6.7%	7.0%
Proposed Method (Tablet format, composite challenge)	3%	12.2%
	5%	8.5%
	10%	4.0%
Proposed Method (Phone format, composite challenge)	3%	16.0%
	5%	9.5%
	10%	4.3%

## Data Availability

The data used in this study are available on request from the corresponding author. The raw data are not publicly available due to these being identifiable biometric data protected by the General Data Protection Regulation (GDPR).
